# Supplementary material for: Sex differences in avian parental care patterns vary across the breeding cycle
Source: Nat Commun. 2023 Nov 1;14:6980. doi: 10.1038/s41467-023-42767-5 (PMC10620184; doi:10.1038/s41467-023-42767-5)
Supplement: Supplementary file 1 — Supplementary Information [file 41467_2023_42767_MOESM1_ESM.pdf]

Supplementary Information:

Sex differences in avian parental care patterns vary across the breeding cycle

Daiping Wang[1,2*], Wenyuan Zhang[3,4], Shuai Yang[1,2], Xiang-Yi Li Richter[5*]

Supplementary Table 1. A list of 15 species with male-only care throughout the breeding cycle.

Scinam_Jetz	English_Jetz	Order	family	builder	incubator	provisoner
Rhea_americana	Greater Rhea	Rheiformes	Rheidae	male	male	male
Rhea_pennata	Lesser Rhea	Rheiformes	Rheidae	male	male	male
Crypturellus_soui	Little Tinamou	Tinamiformes	Tinamidae	male	male	male
Nothoprocta_cinerascens	Brushland Tinamou	Tinamiformes	Tinamidae	male	male	male
Nothura_darwinii	Darwin's Nothura	Tinamiformes	Tinamidae	male	male	male
Dromaius_novaehollandiae	Emu	Casuariiformes	Casuariidae	male	male	male
Pedionomus_torquatus	Plains-wanderer	Charadriiformes	Pedionomidae	male	male	male
Rostratula_australis	Australian Painted Snipe	Charadriiformes	Rostratulidae	male	male	male
Actophilornis_africanus	African Jacana	Charadriiformes	Jacanidae	male	male	male
Hydrophasianus_chirurgus	Pheasant-tailed Jacana	Charadriiformes	Jacanidae	male	male	male
Metopidius_indicus	Bronze-winged Jacana	Charadriiformes	Jacanidae	male	male	male
Jacana_spinosa	Northern Jacana	Charadriiformes	Jacanidae	male	male	male
Jacana_jacana	Wattled Jacana	Charadriiformes	Jacanidae	male	male	male
Phalaropus_lobatus	Red-necked Phalarope	Charadriiformes	Scolopacidae	male	male	male
Phalaropus_fulicarius	Red Phalarope	Charadriiformes	Scolopacidae	male	male	male

Supplementary Table 2. Cross-validation of our data collection with the independent dataset of Cooney et al. (2020) regarding parental care patterns during incubation. P values are derived from two-sided tests.

Compared with Cooney et al. (2020)			
Our dataset	Pearson correlation	Sample size	p-value
species_1533_texts_upload.xlsx	0.8144	991	0
species_1533_texts_SY_upload.xlsx	0.8138	991	0

Supplementary Table 3. Cross-validation of our data collection with the independent dataset of Szekely et al. (2022) regarding parental care patterns during nest building, incubation, and offspring provisioning. Despite the high consistency between the datasets, the agreements during the offspring provisioning stage are slightly lower than during the other two stages. This is because we included the cases where parents of precocial species leading their chicks to food before they reached independence, which was not considered as post-fledging feeding in the dataset of Szekely et al. (2022). P values are derived from two-sided tests.

Nest building, compared with Szekely et al. (2022)			
Our dataset	Pearson correlation	Sample size	p-value
species_1533_texts_upload.xlsx	0.8564	1035	0
species_1533_texts_SY_upload.xlsx	0.8558	1035	0

Incubation, compared with Szekely et al. (2022)			
Our dataset	Pearson correlation	Sample size	p-value
species_1533_texts_upload.xlsx	0.9229	1083	0
species_1533_texts_SY_upload.xlsx	0.9359	1083	0

Offspring provisioning, compared with Szekely et al. (2022)			
Our dataset	Pearson correlation	Sample size	p-value
species_1533_texts_upload.xlsx	0.7297	408	0
species_1533_texts_SY_upload.xlsx	0.6134	408	0
