# Supplementary material for: Sex differences in avian parental care patterns vary across the breeding cycle
Source: Nat Commun. 2023 Nov 1;14:6980. doi: 10.1038/s41467-023-42767-5 (PMC10620184; doi:10.1038/s41467-023-42767-5)
Supplement: Supplementary file 3 — Description of Additional Supplementary Files [file 41467_2023_42767_MOESM3_ESM.pdf]

Supplementary Data 1. Summary of statistics of five linear mixed-effects models (Model 1 to Model 5) based on the dataset compiled by the original author. The coding of response variables in each model is consistent with that in Table 1 of the main text. For the random effect, the size of the variance components is shown. For each fixed effect, the estimate with its standard error (SE), t-value, and corresponding p-value are shown (two-sided tests; no adjustments were made for multiple comparisons).

Supplementary Data 2. Output summary of multivariate phylogenetic models estimating phylogenetic correlations based on the dataset compiled by the original author. Here, the species tree was inversed into a phylogenetic covariance matrix and added as a random effect. The 'phylogenetic signal' of each response variable (grey background) and 'phylogenetic correlations' between these three response variables are shown. The coding of response variables in each model is consistent with that in Table 1 of the main text. For each fixed effect, the estimate with its standard error (SE) and corresponding p-value are shown (two-sided tests; no adjustments were made for multiple comparisons).

Supplementary Data 3. Output summary of five linear mixed-effects models (Model 1 to Model 5) by excluding uncertain species based on the dataset compiled by the original author. The coding of response variables in each model is consistent with that in Table 1 of the main text. For random effects, the size of the variance component is shown. For each fixed effect, the estimate with its SE, the t value, and the corresponding p-value are shown (two-sided tests; no adjustments were made for multiple comparisons).

Supplementary Data 4. Output summary of five linear mixed-effects models (Model 1 to Model 5) by checking the interactions between research effort and sexual selection/the three parental care categories directly. The analysis was based on the dataset compiled by the original author. The coding of response variables in each model is consistent with that in Table 1 of the main text. For random effects, the size of the variance component is shown. For each fixed effect, the estimate with its SE, the t value, and the corresponding p-value are shown (two-sided tests; no adjustments were made for multiple comparisons).

Supplementary Data 5. Summary of statistics of five phylogenetically controlled regression models (Model 1 to Model 5) based on the dataset compiled by the independent author. The coding of response variables in each model is consistent with that in Table 1 of the main text. For the random effect (i.e., the phylogenetic tree), the estimated λ is shown. For each fixed effect, the estimate with its standard error (SE), t-value, and corresponding p-value are shown (two-sided tests; no adjustments were made for multiple comparisons). Note that, for each model, we ran the model using 100 different phylogenetic trees from Jetz et al. (2012). Results are therefore based on mean estimates for predictor slopes and model-averaged standard errors.

Supplementary Data 6. Summary of statistics of five phylogenetically controlled regression models (Model 1 to Model 5) based on the common data entries between two independent datasets compiled by the original author and the independent author, respectively. The coding of response variables in each model is consistent with that in Table 1 of the main text. For the random effect (i.e., the phylogenetic tree), the estimated λ is shown. For each fixed effect, the estimate with its standard error (SE), t-value, and corresponding p-value are shown (two-sided tests; no adjustments were made for multiple comparisons). Note that, for each model, we ran the model using 100 different phylogenetic trees from Jetz et al. (2012). Results are therefore based on mean estimates for predictor slopes and model-averaged standard errors.
