# Supplementary material for: Sex differences in avian parental care patterns vary across the breeding cycle
Source: Nat Commun. 2023 Nov 1;14:6980. doi: 10.1038/s41467-023-42767-5 (PMC10620184; doi:10.1038/s41467-023-42767-5)
Supplement: Supplementary file 5 — Reporting Summary [file 41467_2023_42767_MOESM5_ESM.pdf]

nature portfolio

Corresponding author(s): Daiping Wang; Xiang-Yi Li Richter

Last updated by author(s): Sep 29, 2023

Reporting Summary

Nature Portfolio wishes to improve the reproducibility of the work that we publish. This form provides structure for consistency and transparency in reporting. For further information on Nature Portfolio policies, see our Editorial Policies and the Editorial Policy Checklist.

Statistics

For all statistical analyses, confirm that the following items are present in the figure legend, table legend, main text, or Methods section.

n/a	Confirmed	
☐	☒	The exact sample size (*n*) for each experimental group/condition, given as a discrete number and unit of measurement
☐	☒	A statement on whether measurements were taken from distinct samples or whether the same sample was measured repeatedly
☐	☒	The statistical test(s) used AND whether they are one- or two-sided<br>*Only common tests should be described solely by name; describe more complex techniques in the Methods section.*
☐	☒	A description of all covariates tested
☐	☒	A description of any assumptions or corrections, such as tests of normality and adjustment for multiple comparisons
☐	☒	A full description of the statistical parameters including central tendency (e.g. means) or other basic estimates (e.g. regression coefficient) AND variation (e.g. standard deviation) or associated estimates of uncertainty (e.g. confidence intervals)
☐	☒	For null hypothesis testing, the test statistic (e.g. *F*, *t*, *r*) with confidence intervals, effect sizes, degrees of freedom and *P* value noted<br>*Give P values as exact values whenever suitable.*
☐	☒	For Bayesian analysis, information on the choice of priors and Markov chain Monte Carlo settings
☐	☒	For hierarchical and complex designs, identification of the appropriate level for tests and full reporting of outcomes
☐	☒	Estimates of effect sizes (e.g. Cohen's *d*, Pearson's *r*), indicating how they were calculated

Our web collection on statistics for biologists contains articles on many of the points above.

Software and code

Policy information about availability of computer code

Data collection	Birds of the World database:Birds of the World. Cornell Laboratory of Ornithology, Ithaca, NY, USA. (2020). DOI: https://birdsoftheworld.org/bow/home. All datasets generated and analyzed in the current study, and relevant code for analyses and plotting are available in the online repository: https://osf.io/g4xra/. Source data are provided as a Source Data file.
Data analysis	All analyses were carried out within R statistical environment (2022) including packages 'MCMCglmm', 'lme4' and'phylolm' .All datasets generated and analyzed in the current study, and relevant code for analyses and plotting are available in the online repository: https://osf.io/g4xra/. Source data are provided as a Source Data file.

For manuscripts utilizing custom algorithms or software that are central to the research but not yet described in published literature, software must be made available to editors and reviewers. We strongly encourage code deposition in a community repository (e.g. GitHub). See the Nature Portfolio guidelines for submitting code & software for further information.

Data

Policy information about availability of data

All manuscripts must include a data availability statement. This statement should provide the following information, where applicable:

- Accession codes, unique identifiers, or web links for publicly available datasets
- A description of any restrictions on data availability
- For clinical datasets or third party data, please ensure that the statement adheres to our policy

All datasets generated and analyzed in the current study, and relevant code for analyses and plotting are available in the online repository: https://osf.io/g4xra/. Source data are provided as a Source Data file.

Research involving human participants, their data, or biological material

Policy information about studies with human participants or human data. See also policy information about sex, gender (identity/presentation), and sexual orientation and race, ethnicity and racism.

Reporting on sex and gender	This information has not been collected.
Reporting on race, ethnicity, or other socially relevant groupings	See above
Population characteristics	See above
Recruitment	See above
Ethics oversight	See above

Note that full information on the approval of the study protocol must also be provided in the manuscript.

Field-specific reporting

Please select the one below that is the best fit for your research. If you are not sure, read the appropriate sections before making your selection.

☐ Life sciences ☐ Behavioural & social sciences ☒ Ecological, evolutionary & environmental sciences

For a reference copy of the document with all sections, see nature.com/documents/nr-reporting-summary-flat.pdf

Ecological, evolutionary & environmental sciences study design

All studies must disclose on these points even when the disclosure is negative.

Study description	A large comparative study using species data in birds. We surveyed all bird species (n = 10721 species) in the Birds of the World database. For each species, we focused on its breeding section for the further data collection (details see Methods of our manuscript).
Research sample	We surveyed all bird species (n = 10721, most complete data set regarding the breeding records) in the breeding section from the Birds of the World database. For each species, we collected which sex provides parental care in each of the three forms — nest building, incubation, and offspring provisioning — across a reproductive cycle.
Sampling strategy	For all bird species (n = 10721), we collected 1533 species which have the information regarding which sex provides parental care in each of the three forms — nest building, incubation, and offspring provisioning — across a reproductive cycle. This is the largest data set we can get so far.
Data collection	We took notes of the parental care features for each species from the breeding section of the species' account and then classified them into four categories for each form of care. We have the original observer and another independent observer who collected the same data set.
Timing and spatial scale	Data collection carried out from 2021-2022.
Data exclusions	In rare cases (N=49 species), the parental care information was recorded with uncertain words, such as "reportedly" or "probably" in one or more care forms (e.g., White-throated Bulbul: nest reportedly built by both sexes; ... incubation possibly by both sexes, period 13 days; chicks fed by both parents). All statistical models were run by first including and then excluding those uncertain data.
Reproducibility	We used different statistical methods and models to analyze data. Results from these different models are consistent. Further, results based on the data collected by the original observer are consistent with results based on the data collected by another

	independent observer (see more details from the article).
Randomization	For all bird species (n = 10721), we set up objective sampling rules and procedures before data collections. Following the procedures, we collected 1533 species with 'full data' (i.e., information about sex roles in all three care forms; 651 non-passerines and 882 passerine species). These 1533 species which have the information regarding which sex provides parental care in each of the three forms — nest building, incubation, and offspring provisioning — across a reproductive cycle. This is the largest data set we can get so far.
Blinding	Data collection followed sampling rules and procedures as stated above. We got the laegest data set (n = 1533). The collated datasets were blinded before statistical tests. Further, the data set collected by the independent observer are totally blinded before statistical tests. Results based on the data collected by the original observer are consistent with results based on the data collected by another independent observer (see more details from the article).

Did the study involve field work? ☐ Yes ☒ No

Reporting for specific materials, systems and methods

We require information from authors about some types of materials, experimental systems and methods used in many studies. Here, indicate whether each material, system or method listed is relevant to your study. If you are not sure if a list item applies to your research, read the appropriate section before selecting a response.

Materials & experimental systems

n/a	Involved in the study
☒	☐ Antibodies
☒	☐ Eukaryotic cell lines
☒	☐ Palaeontology and archaeology
☒	☐ Animals and other organisms
☒	☐ Clinical data
☒	☐ Dual use research of concern
☒	☐ Plants

Methods

n/a	Involved in the study
☒	☐ ChIP-seq
☒	☐ Flow cytometry
☒	☐ MRI-based neuroimaging
